# Histopathological Experience of Primary Adrenal Lymphoma From Two Tertiary Hospitals

**DOI:** 10.7759/cureus.42940

**Published:** 2023-08-04

**Authors:** Jaudah Al-Maghrabi

**Affiliations:** 1 Department of Pathology, Faculty of Medicine, King Abdulaziz University, Jeddah, SAU; 2 Department of Pathology and Laboratory Medicine, King Faisal Specialist Hospital & Research Center, Jeddah, SAU

**Keywords:** saudi arabia, primary, histopathology, adrenal gland, lymphomas

## Abstract

Introduction: Primary adrenal lymphoma (PAL) is a rare tumor. The aim of this study was to demonstrate the histopathological features of PAL at two tertiary hospitals.

Materials and methods: All PALs diagnosed between January 2003 and February 2023 were retrieved. Pathology and immunohistochemistry slides were reviewed. Additional immunohistochemical markers were done in selected cases. Follow-up data were obtained.

Results: There were 7 cases of PAL. The age range of the patients was 52 to 73 years (median 64 years; mean 63.3 years). There were 4 males (57.1%) and 3 females (42.9%). The clinical manifestations included abdominal pain nausea, vomiting, and loss of weight. There were 4 cases of diffuse large B-cell lymphoma (DLBCL), 2 cases of high-grade B-cell lymphomas, and 1 case of follicular lymphoma. There were 5 cases that were unilateral and 2 cases that were bilateral, and both were high-grade B-cell lymphoma. During follow-up, the 1-year and 2-year overall survival rates were 50% and 33%, respectively.

Conclusion: PAL is a disease of the elderly, and DLBCL is the most common pathological type. The prognosis is generally poor. Further reporting of PAL cases might help in understanding this disease and could lead to improvement in its management.

## Introduction

Lymphoid neoplasms that arise primarily in the adrenal glands are extremely rare, and information is derived from small sample studies. These tumors are critical to diagnosing and differentiating from other adrenocortical malignant neoplasms because of the different management. Secondary adrenal involvement in lymphoma is rare, and the occurrence rate is 0.2-5% [1, 2]. Primary adrenal lymphomas (PALs) are very rare neoplasms and account for less than 1% of non-Hodgkin’s lymphomas [3, 4]. The clinicopathological pattern of PAL in Saudi patients is not well described and the data are limited to a few case reports [5-8]. Thus, more clinicopathological detail is needed to better understand this disease. The aim of the study was to review the histopathological features of all PAL cases diagnosed at two tertiary institutions.

## Materials and methods

The study was carried out at two referral institutions in Western Saudi Arabia, the King Abdulaziz University Hospital (KAUH) and King Faisal Specialist Hospital and Research Centre (KFSHRC). The study included all lymphomas diagnosed in the adrenal glands including the referral pathology consultations. The definition proposed by Krol et al. was used to define primary extranodal non-Hodgkin's lymphoma (NHL), which includes all patients who present with NHL originating at the adrenal glands, even in the presence of disseminated disease if the adrenal component is clinically dominant [9].

The collected clinical data included age at presentation, gender, clinical features, treatment, and outcome. Immunohistochemistry slides were reviewed, and more immunohistochemistry marker tests were performed in selected cases. The minimum immunohistochemistry panel included CD45, CD20, CD3, BCL-2, BCL-6, CD10, MUM-1, and KI-67. Additional panels were added in selected cases and included pankeratin EMA, melan-A, synaptophysin, chromogranin, CD56, S100, and calretinin.

The archival material that was used in the study included formalin-fixed paraffin-embedded (FFPE) material following fixation by 10% neutral buffered formalin (NBF). Blocks of neoplasms were cut at 4 µm. The sections were mounted on positive-charged slides (Leica Microsystems Plus Slides), and then, were deparaffinized in xylene and rehydrated in an automated immunostainer (BenchMark XT, Ventana® Medical Systems Inc., Tucson, AZ, USA). Pre-treatment was done using CC1 (prediluted cell conditioning solution) for 60 min. The additional antibodies were used as per company recommendation. Negative and positive control slides were included. Immunostaining was reported as positive or negative.

Histopathological classification of lymphomas was done according to the 2017 World Health Organization criteria [10]. Diffuse large B-cell lymphomas (DLBCLs) were subclassified according to the Hans algorithm for germinal center B-cells (GCB) and non-germinal center B-cells (non-GCB), which depends on the pattern of immunohistochemical expression for CD10, MUM-1, and BCL-6 [11].

The study covered the cases diagnosed between January 2003 and February 2023. The study was approved (Reference No. 34-22) by the Research Committee of the Biomedical Ethics Unit at the Faculty of Medicine, King Abdulaziz University, Jeddah, Saudi Arabia. A review of morphology and additional immunohistochemistry markers allowed for the re-classification of older cases into currently accepted diagnostic categories.

## Results

Seven cases of PAL were identified. Clinical and histopathological data are summarized in Table 1.

**Table 1:** Summary of the PAL cases diagnosed at KAUH and KFSHRC, Jeddah -ve: Negative N/A: Not available BM: Bone marrow CT: Computed tomography DLBCL: Diffuse large B-cell lymphoma R-CHOP: Rituximab, cyclophosphamide, doxorubicin, vincristine, and prednisolone R-ESHAP: Rituximab, etoposide, solu-medrone, high dose Ara C, cisplatin PAL: Primary adrenal lymphoma KAUH: King Abdulaziz University Hospital KFSHRC: King Faisal Specialist Hospital and Research Centre

	Age/Sex	Site of involvement	Clinical presentation	Diagnosis	BM	Radiological findings	Tumor size	Follow-up	Treatment /outcome
1	65/male	Left adrenal also involves the lung and spleen	Left flank pain nausea, and weight loss	Follicular lymphoma, grade 2.	-ve	CT: showed enhancing solid mass of left adrenal	5.5 cm	9 months died	Five cycles of obinutuzumab with bendamustine
2	52/female	Bilateral adrenal masses	Abdominal pain nausea, vomiting, and loss of weight	High-grade large-cell lymphoma	-ve	CT: showed a large heterogenous lesion located superior to the right kidney (adrenal) and enhancing mass in the left adrenal and pelvis	11 cm (right adrenal) and 6 cm (left adrenal)	10 months and died	Failed multiple chemotherapy lines: R-CHOP, high-dose methotrexate, R-ESHAP, polatzumab palliative radiotherapy
3	64/male	Left adrenal	Abdominal pain and fever	DLBCL	N/A	CT: showed heterogenous lesion of the left adrenal	6 cm	17 months	R-CHOP
4	73/male	Right adrenal mass	Right flank pain and loss of weight	DLBCL	-ve	CT: showed heterogeneous enhancing solid soft tissue left adrenal mass	5 cm	42 months	R-CHOP
5	66/ female	Left adrenal mass	Nausea, vomiting	DLBCL		CT: showed enhancing solid mass of right adrenal	7 cm	N/A	No available clinical follow-up data
6	62/male	Right adrenal mass	Right flank pain	DLBCL	-ve	CT: showed enhancing solid right adrenal mass	5 cm	32 months	R-CHOP
7	61/male	Bilateral adrenal masses	Abdominal discomfort and weakness	High-grade large-cell lymphoma	-ve	CT: showed homogeneous enhancing bilateral adrenal masses with para-aortic lymph node enlargement	6.5 and 6 cm	1 month and died	Patient had progressive disease and died before any initiation of chemotherapy

The age of the patients ranged between 52 and 73 years (median 64 years; mean 63.3 years), and most of the patients were above the age of 60 years (6 of 7 patients). There were 4 males (57.1%) and 3 females (42.9%). The clinical manifestations were nonspecific and included abdominal pain nausea, vomiting, and loss of weight. Radiological evaluation by enhanced computed tomography (CT) scan indicated solid masses that ranged in size between 5 and 11 cm. The diagnosis was made by needle core biopsy in 6 cases and by surgical adrenalectomy resection in 1 case. 

**Figure 1:**
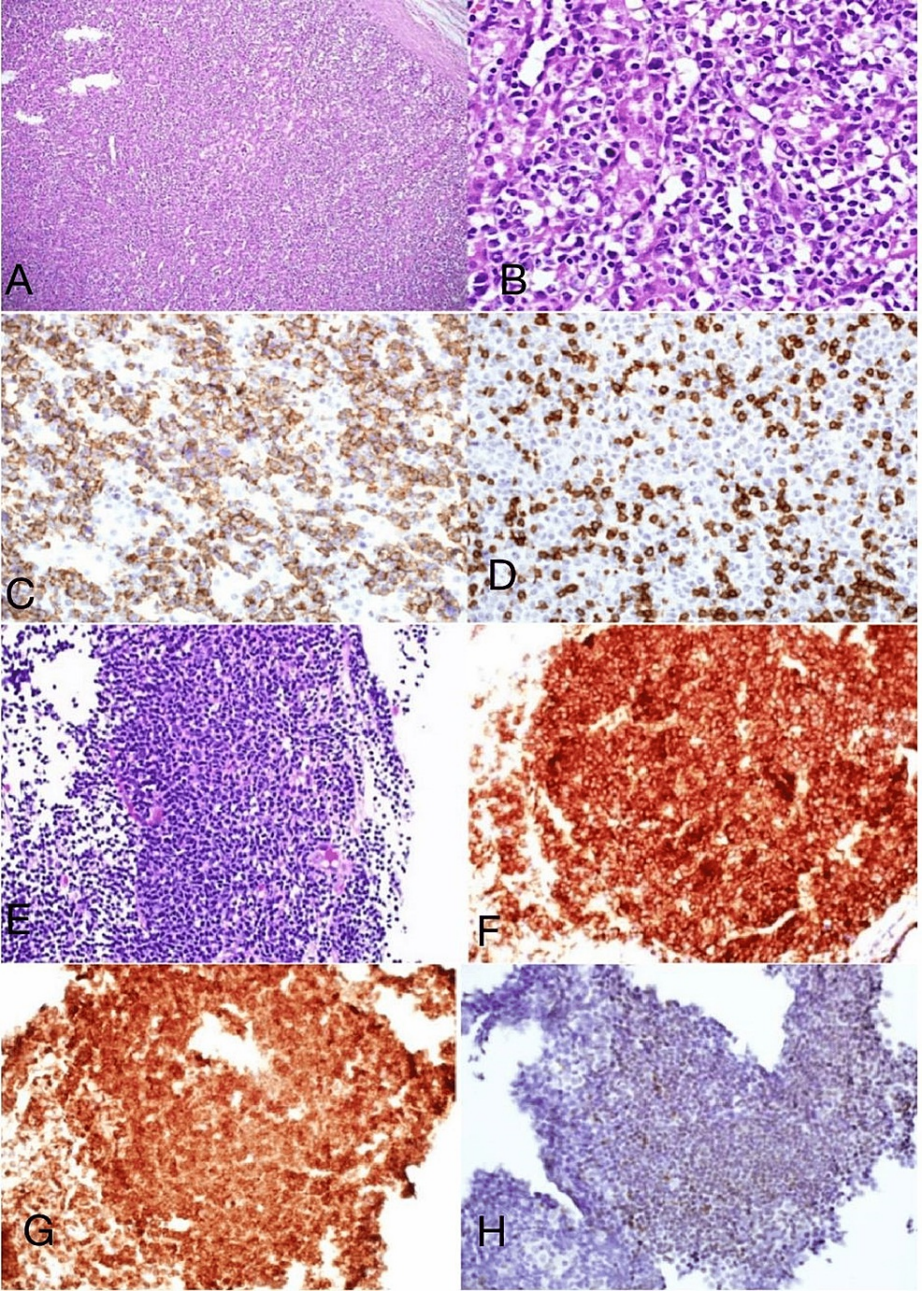
Histopathology of PAL A: Section of the adrenal mass showing diffuse infiltration of large lymphoid cells (Hematoxylin and eosin, 200×) B: Higher power of the same case; reveals large lymphoma cells surrounding adrenal cortical cells (Hematoxylin and eosin, 400×) C: Diffuse large B-cell lymphoma expressing CD20 (Immunohistochemistry stain, 200×) D: Diffuse large B-cell lymphoma with negative staining for CD3, which highlighted reactive T-cells (Immunohistochemistry stain, 200×) E: Section of adrenal mass biopsy showing follicular lymphoma revealing nodular pattern and composed of mixed large and small sized lymphoid cells (Hematoxylin and eosin, 400×) F: Follicular lymphoma expressing CD20 (Immunohistochemistry stain, 200×) G: Follicular lymphoma bcl-2 (Immunohistochemistry stain, 200×) H: Follicular lymphoma bcl-6 (Immunohistochemistry stain, 200×) PAL: Primary adrenal lymphoma

Histopathological evaluation revealed that all cases were non-Hodgkin’s lymphomas There were 4 cases of DLBCL, 2 cases of high-grade B-cell lymphomas of Burkitt-like large cell type, and 1 case of low-grade B-cell follicular lymphoma (Figure 1). The proliferative index by Ki-67 in the DLBCL cases ranged between 50% and 70%. Ki-67 was 15 in the low-grade follicular lymphoma case. Both high-grade lymphomas show a proliferative index of more than 90%.

There were 5 cases that were unilateral and 2 cases that were bilateral. The two bilateral lymphomas were high-grade B-cell lymphoma. All DLBCLs were further subclassified as the non-GCB subtype.

Three of the patients with DLBCL were treated with standard therapy. They received rituximab, cyclophosphamide, doxorubicin, vincristine, and prednisolone (R-CHOP). The fourth patient went to another institution, and there no clinical follow-up data were available. The patient with follicular lymphoma was treated with five cycles of obinutuzumab with bendamustine. Multiple chemotherapy lines failed for one of the patients with high-grade B-cell lymphoma, including R-CHOP, rituximab, etoposide, solu-medrone, high dose Ara C, cisplatin (R-ESHAP), R-BeGiv, and polatzumab. The patient finally received palliative radiotherapy but passed away. The other patient with high-grade B-cell lymphoma had progressive disease and died before any initiation of chemotherapy. The median follow-up duration was 22 months (range: 1 to 42 months). The 1-year and 2-year overall survival rates during follow-up were 50% and 33%, respectively.

## Discussion

PAL is an extremely rare disease and is usually aggressive with a poor prognosis. There are less than 300 cases described in the English literature to date. The information on this entity is still limited to case reports and a few case series reported from India [12], China [2, 13-15], Europe, and North America [16]. There were only four PAL cases previously reported from Saudi Arabia, and all were NHL [5-8] and their clinicopathological data are summarized in Table 2. 

**Table 2:** Summary of the PAL cases reported from Saudi Arabia -ve: Negative N/A: Not available CT: Computed tomography DLBCL: Diffuse large B-cell lymphoma R-CHOP: Rituximab, cyclophosphamide, doxorubicin, vincristine, and prednisolone CVP: Cyclophosphamide, oncovin (vincristine), and prednisolone PAL: Primary adrenal lymphoma

Reference	Age/Sex	Site	Clinical presentation	Diagnosis	Bone marrow	Radiological findings	Treatment/outcome
Ekhzaimy & Mujamammi [6]	55/M	Bilateral large adrenal masses	Flank pain, nausea, vomiting, and weight loss	DLBCL	-ve	CT: showed bilateral, heterogeneous enhanced adrenal masses, measuring 10.6 cm and 7 cm in maximum dimension	Treated with R-CHOP, with partial response
Bedaiwi et al. [5]	71/M	Left adrenal mass	Anemia and weight loss, gradual, painless decline in his vision in both eyes related to ocular metastasis	T-cell lymphoma	-ve	CT: revealed a large heterogeneous mass involving the left adrenal gland with suspicious invasion	Treated with CVP. After two cycles of chemotherapy, he deteriorated and passed away
Al Shareef et al. [7]	58/ F	Bilateral large adrenal masses	Abdominal pain, loss of appetite, loss of weight, fever, and night sweating	DLBCL	N/A	CT: showed homogeneously enhanced bilateral adrenal masses (7.5 and 4.9 cm), with large para-aortic lymph node enlargement	Patient's condition deteriorated further and she passed away
Ghareeb et al. [8]	62/F	Bilateral large adrenal enlargement	fatigue, weight loss, fever, and skin discoloration	DLBCL	N/A	CT: revealed bilateral adrenal enlargement	Patient showed quick disease progression and passed away after a short period of the diagnosis, before the initiation of chemotherapy

The age of the patients in the current study ranged between 52 and 73 years. In the previously reported cases from Saudi Arabia, the patients ranged between 55 and 71 years old and included 2 males and 2 females. Three of those cases were DLBCL, and one case was T-cell lymphoma. Three of those cases were bilateral, two were treated with chemotherapy, and two deteriorated before initiation of chemotherapy

According to the data obtained from Surveillance, Epidemiology, and End Results (SEER), Li et al. showed that DLBCL is the most common pathological type [17]. Primary adrenal DLBCL predominantly affects patients who are male, white, and elderly [17]. Age above 70 years, bilaterality, and chemotherapy treatment are recognized as prognostic factors that have a correlation with adverse overall and cause-specific survivals [17]. No specific standard therapeutic protocol for PAL has yet been established. However, since DLBCL is the most common pathological type of PAL, the standard therapy for DLBCL, including CHOP or R-CHOP, has been used in the treatment of most reported cases of PAL. Nevertheless, chemotherapy could only achieve remission in about one-third of PAL patients [18].

Surgical resection has a limited value in the treatment of this tumor [18]. The most frequent pathological subtype (70%) of PAL is non-GCB type DLBCL [2]. All DLBCLs in the current series were subclassified as the non-GCB subtype. Most of the reported cases of PAL worldwide were DLBCL. In the literature, other histopathological types of PAL have also been described, including Burkitt-like large-cell lymphoma [19], lymphoblastic [20], Mantle cell [21], anaplastic [22], T-cell lymphoma [5, 23], follicular lymphoma [24], Hodgkin’s lymphoma [25], and intravascular large B-cell lymphoma (IVLBCL) [26, 27]. PAL generally has a dismal prognosis; however, early diagnosis and immediate therapy with a rituximab-containing regimen combined with prophylactic intrathecal chemotherapy can improve the prognosis [2].

Bilateral adrenal neoplasms are uncommon. Adrenal glands are frequently involved with metastasis in patients with different types of carcinomas. The most common primary sites of metastasis to the adrenals are the lungs, breasts, and melanoma [28]. Bilateral metastases are more common than unilateral metastatic tumors [28]. The bilateral pattern is seen in about 70% of PAL cases according to some studies [2]. Two of 7 patients in this series and 3 of the 4 previously reported cases from Saudi Arabia showed bilateral adrenal involvement. Other adrenal tumors that tend to be bilateral include malignant pheochromocytomas and metastasis. Adrenocortical adenomas and carcinomas are usually unilateral. Because of the relatively high possibility of bilaterality of PAL, it seems reasonable to do a biopsy in those patients after ruling out pheochromocytoma using a biochemical study.

Zhou et al. found that primary adrenal lymphoma is the second most common cause of bilateral adrenal neoplasm after pheochromocytoma [29]. They showed that non-functioning cortical adenoma and metastatic tumors rank third and fourth, respectively. They also showed that PAL can be distinguished from other adrenal tumors based on older age at presentation, higher frequency in males, and larger neoplasm (6 cm) [29]. Our results are in concordance with these findings regarding PAL characteristics except for the gender of the patients in that there was no significant difference between males and females. Six of the seven patients in the current study were above the age of 60 years, and all the cases showed tumor mass of size more than 5 cm, including 4 cases with size more than 6 cm.

The pathogenesis of PAL is unclear. Histologically, normal adrenal glands do not contain lymphoid tissue. PAL most likely arises from lymphoid tissue that develops in the background of adrenal chronic inflammation or possibly by the spread of lymphoma cells from an involved contralateral adrenal gland. Suggested theories on the pathogenesis of PAL include the presence of autoimmune adrenalitis, immune dysfunction, viral infection such as human immunodeficiency virus (HIV) and Epstein virus (EBV), and genetic mutation of p53 and the c-kit gene [30-32]. The homing theory of hematopoietic tissue may explain the bilateral adrenal lymphomas with a lack of bone marrow and nodal involvement [33]. Rarely, lymphoproliferative disorders can develop in the adrenal gland as a result of immunosuppressive therapy for organ transplants [34-38] or other diseases [39].

The prognosis of PAL is generally poor, and the 1-year and 2-year overall survival rates in the current study were 50% and 33%, respectively. Two of the previously reported cases from Saudi Arabia died before the initiation of chemotherapy, one died after 3 cycles of chemotherapy, and one patient showed partial response to chemotherapy. According to SEER data [17], the overall survival rates of patients with DLBCL at 5 and 10 years were 19.17% and 3.33%, respectively. In one study with a longer follow-up period, the estimated 5-year overall survival was around 50% [2].

Several cases of PALs with central nervous system (CNS) involvement have been reported and showed a male predominance (94.5% were male), and all the patients (100%) had bilateral PAL [40]. PAL with synchronous involvement of the hypothalamic region has also been reported [24]. PAL has been reported in association with other adrenal neoplasms such as pheochromocytoma [41].

## Conclusions

PAL is a disease of the elderly. DLBCL is the most common pathological type. The prognosis is generally poor. Clinical presentations are abdominal pain, nausea, vomiting, and loss of weight. Because of the high possibility of bilaterality in PAL, it seems reasonable to do a biopsy for those patients. Further reporting of PAL cases might help in understanding this disease and could lead to improvement in its management.
